# Ewing sarcoma of the temporal bone with aneurysmal bone cyst-like changes: A rare case report with an unusual radiological presentation

**DOI:** 10.1177/19714009231212358

**Published:** 2023-11-03

**Authors:** Isabel Lloret, Ivar Hompland, Ingvild VK Lobmaier, Jarle Sundseth, Andres Server

**Affiliations:** 1 Department of Radiology, The Norwegian Radium Hospital, 155272 Oslo University Hospital, Norway; 2 Department of Oncology, The Norwegian Radium Hospital, 155272 Oslo University Hospital, Norway; 3 Department of Pathology, The Norwegian Radium Hospital, 155272 Oslo University Hospital, Norway; 4 Department of Neurosurgery, Rikshospitalet, 155272 Oslo University Hospital, Norway; 5 Section of Neuroradiology, Department of Radiology, Rikshospitalet, 155272 Oslo University Hospital, Norway

**Keywords:** Ewing sarcoma, temporal bone, skull, MRI, CT

## Abstract

Ewing sarcoma (ES) is a malignant small round cell tumor, accounting for 10–15% of all primary bone tumors and approximately 3% of all pediatric cancers. Primary ES of the cranial bone is unusual with reported incidence from 1% to 6% of all ES cases. This report shows a rare case of primary ES of the squamous temporal bone in a 12-year-old boy with a history of swelling of the right temporal region and symptoms of increased intracranial pressure. We illustrate the extremely unusual radiological presentation of this primary ES of temporal bone associated with large aneurysmal bone cyst-like (ABC-like) changes. The boy was successfully treated according to Euro Ewing 2012 protocol. He is alive with no evidence of recurrence and metastasis after 16 months of completed treatment.

## Introduction

In 1921, James Ewing (1866–1943) described a round cell sarcoma of the bone occurring in children and young adults that he called diffuse endothelioma.^1^ Ewing sarcoma (ES) is a malignant small round cell tumor associated with structural rearrangements that generate FET-ETS fusion genes, creating an aberrant transcription factor.^2^ Following osteosarcoma, ES is the second most common malignant primary bone tumor of children and adolescents. The tumor is generally diagnosed during the second decade of life and is lightly more prevalent in males. Primary ES of the cranial bones is rare, accounting for roughly 1–6% of all ES cases.^2–4^

A multimodality imaging approach is used in detection and follow-up. Imaging features of osseous ES often suggest the diagnosis. The typical imaging is a moth-eaten to permeative bone destruction with poor margination. The tumor is usually associated with a large homogeneous or mildly heterogeneous soft-tissue mass that is related to the high degree of cellularity.^5,6^ Aneurysmal bone cyst-like (ABC-like) areas can be seen in bone tumors that have undergone hemorrhagic cystic changes. Most of the ABC-like changes are associated with giant cell tumor, osteoblastoma, chondroblastoma, fractured bone cyst, and telangiectatic osteosarcoma.^7^ ABC-like areas are exceptionally rare in osseous ES and most common seen after treatment.^5^

The purpose of this report is to illustrate the extremely rare radiological presentation of this ES of the temporal bone. The present study provides data regarding the clinical presentation, pathological characteristics, and therapeutic management.

## Case report

A 12-year-old boy was presented with a history of swelling in the right temporal region for the past 3–4 months. The patient also had increasing lethargy and headache almost every morning as well as nausea with vomiting. No fever or weight loss. No ear infections or vision loss.

Patient was admitted to a local hospital and examined with magnetic resonance imaging (MRI) and computed tomography (CT). MRI showed a mass in the squamous part of right temporal bone with a small extracranial soft-tissue component and a large intracranial soft-tissue component, causing mass effect and a displacement of the brain structures. The small extracranial component had solid appearance (Figure 1), homogeneous intermediate T2-signal intensity, contrast enhancement, and high signal intensity on diffusion-weighted imaging (DWI) with a corresponding low apparent diffusion coefficient value (ADC: 0.7 × 10^−3^ mm^2^/s), indicating high tumor cellularity. However, the large intracranial component mainly contained multi-cystic spaces of variable size with fluid–fluid levels (Figure 1), surrounded by a rim of low T1 and T2 signal intensity, and smooth septal and rim contrast enhancement, regarded as ABC-like changes, which is a very rare and atypical presentation in ES. Focal areas of solid component near temporal bone were also seen. CT showed an ill-defined, permeative bone destruction in the squamous part of the right temporal bone; furthermore, an aggressive periosteal reaction with spiculated pattern and expanded cortex with Codman triangle was identified (Figure 1).

**Figue 1.**
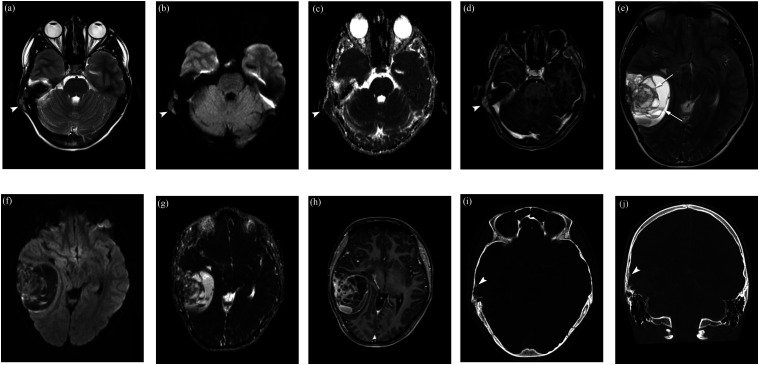
Multiparametric magnetic resonance imaging (MRI) shows a tumor arising from the squamous part of right temporal bone with a small extracranial soft-tissue mass and a large intracranial soft-tissue mass compressing the brain structures. The small extracranial soft-tissue mass (white arrowhead) reveals homogeneous intermediate signal intensity on axial T2W imaging (a), high signal intensity on axial diffusion-weighted imaging (DWI, b = 1000 s/mm^2^) with a corresponding low apparent diffusion coefficient value (ADC: 0.7 × 10^−3^ mm^2^/s) (b), (c), and homogeneous contrast enhancement on postgadolinium axial T1W imaging (d). The large intracranial soft-tissue mass mainly contains blood-filled spaces of variable size with fluid–fluid levels on T2W, axial DWI (b = 1000 s/mm^2^), and ADC images (e)–(g), and smooth septal and rim contrast enhancement (h), suggesting ABC-likes changes (white arrows). Axial and coronal CT (i), (j) images show an ill-defined, permeative bone destruction in the squamous part of right temporal bone. Aggressive spiculated periosteal reaction and expanded cortex with Codman triangle (white arrowhead).

**Figure 2.**
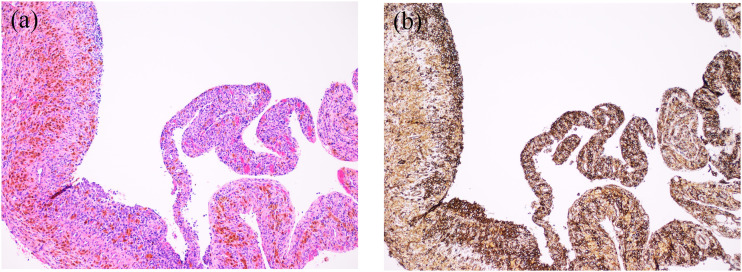
(a) H&E (×100) staining showing cystic appearing areas with rest of vital small round cells. (b) Immunohistological staining with CD99 confirming that the round cells represent rest of Ewing sarcoma.

The biopsy specimen of the extracranial component showed an undifferentiated small round cell neoplasm. Perivascular pseudorosettes were not identified. We performed immunohistochemical analysis with CD99 that is a cell-surface relevant glycoprotein and a relevant diagnostic marker for Ewing sarcoma, showing diffuse strong membranous expression of CD99. Furthermore, we used fluorescence in-situ hybridization (FISH) showing EWS split, and the diagnosis of ES was confirmed with NGS (Oncomine Childhood Cancer panel-Thermo Fisher), showing EWSR1-FLI1 fusion (Figure 2).

There were no signs of metastasis on chest CT. Whole-body ^18^fluoro-2-deoxy-D-glucose positron emission tomography/computed tomography (^18^F-FDG-PET/CT) revealed a mild high tracer uptake in the small extracranial tumor component, and almost lack of uptake of the large intracranial component in the right temporal region. There were no signs of distant bone metastasis.

After diagnosis, the patient started neoadjuvant chemotherapy including vincristine, doxorubicin, cyclophosphamide, ifosfamide, and etoposide according to Euro Ewing 2012 protocol.^8^

**Figure 3.**
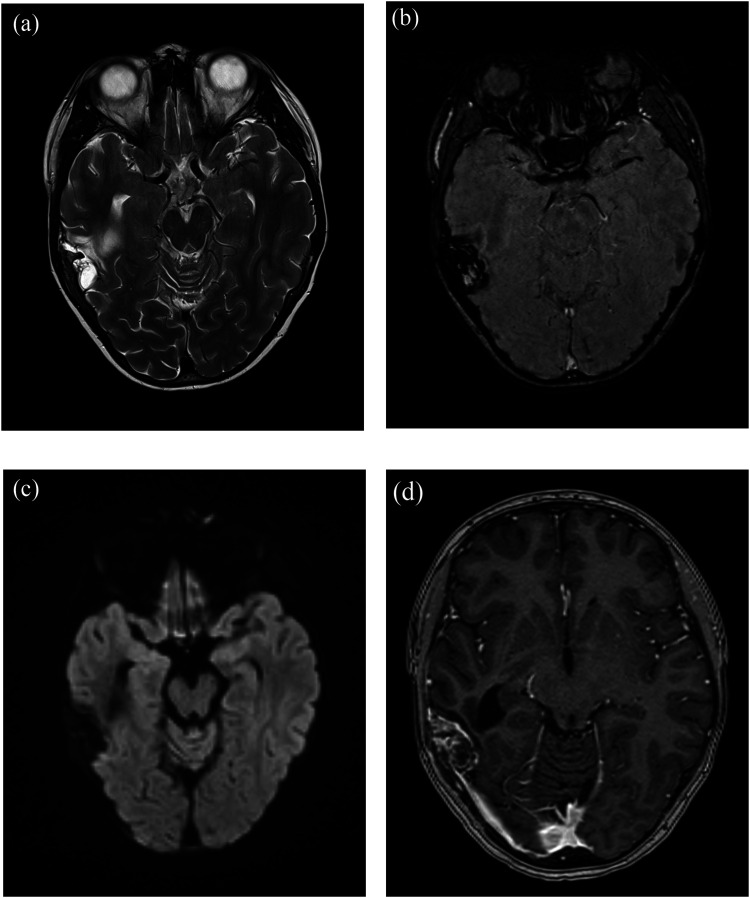
Follow-up MRI, 4 months after the initiation of neoadjuvant chemotherapy and before surgery, shows full regression of extracranial soft-tissue mass and partial regression of the intracranial soft-tissue mass. The intracranial component showed areas reminiscent of ABC-likes changes (blood-filled spaces with hemosiderin contain) on axial T2W imaging (a) and on axial susceptibility weighted imaging (SWI) (b). No areas of high signal intensity on axial DWI (b = 1000 s/mm^2^) (c), and septal and rim contrast enhancement near temporal bone on axial postgadolinium T1W imaging (d), indicating some viable residual tumor tissue.

The follow-up MRI of the head performed 2 months (4 chemotherapy cycles) and 4 months (9 chemotherapy cycles) after the initiation of neoadjuvant chemotherapy showed complete response of the small extracranial soft-tissue component and partial response of the intracranial soft-tissue component, indicating a good treatment response. The intracranial component showed areas reminiscent of ABC-likes changes, no areas of low diffusion, and septal and rim contrast enhancement, indicating some viable residual tumor tissue (Figure 3).

The patient underwent radical surgery after 9 chemotherapy cycles. The surgical procedure was performed under general anesthesia, with the patient in the supine position, and the head placed in a Mayfield_®_ scull clamp (Integra LifeSciences). The Brainlab™ neuronavigation system (Brainlab AG) was used to localize the tumor and venous sinuses for optimal planning of the craniotomy. A horseshoe-shaped skin incision was placed from the front of the ear to the sigmoid sinus. The muscular fascia and periosteum showed suspected tumor infiltration and were excised. A temporal/subtemporal craniotomy was performed, visualizing the dura, which was tumor infiltrated, as well as the tabula interna of the removed cranial bone flap, making it necessary to plan for duroplasty and cranioplasty. Suspected tumor infiltration of the basal temporal bony edges towards the mastoid cells was removed using a high-speed drill (Midas Rex™ Medtronic), and the mastoid cells closed with bone wax. The infiltrated dura was then widely excised. A Leica microscope (Leica Microsystems) was introduced, and the tumor followed circumferentially towards the borders of normal brain parenchyma, the middle fossa, and the tentorium. A gross total tumor resection was achieved. The durotomy and dural defect were rectified using a non-synthetic dura substitute (Durepair™ Regeneration Matrix, Medtronic). The tumor infiltrated skull bone was replaced using a Porous High-Density Polyethylene implant (Omnipore® Matrix Surgical) to close the craniotomy.

After neoadjuvant chemotherapy and surgery, histopathologic analysis showed extensive remaining vital tumor tissue. Some of the tumor tissue formed cyst wall fragments (Figure 2).

After surgery, local proton beam radiation (54 Gy RBE in 1.8 Gy daily fractions with a boost 5.4 RBE in 1.8 daily fractions to osteolytic lesion) and 5 adjuvant chemotherapy cycles completed the treatment. At 16-month follow-up, the patient was doing well, with no signs of tumor recurrence or metastasis.

## Discussion

ES is a malignant small round cell tumor, accounting for 10–15% of all primary bone tumors and approximately 3% of all pediatric cancers. Most cases of osseous ES arise in the pelvis, axial skeleton and in the diaphysis or metadiaphysis of long bones.^5^ Primary ES of the cranial bone is rare, with reported incidence from 1% to 6% of all ES.^3,4,9^ The most involved cranial locations are the frontal and temporal bone.^10^ Multidisciplinary management is the mainstay of an accurate diagnosis and successful treatment of ES of the cranial bones.

Clinical presentation of ES of the cranial bones is diverse and related to the site of involvement and aggressiveness. The most common symptom is a tender, expansive mass, and signs of increased intracranial pressure such headache, visual deficit, and vomiting.^9,10^

A multimodality imaging approach is used in detection and follow-up of ES. CT and MRI play a crucial role in forming a differential diagnosis and determinate the extent of disease, treatment planning, and long-term management.

Imaging features of osseous ES often suggest the diagnosis, but it can be challenging as we show in our case. At CT, the typical imaging of ES is bone destruction with a moth-eaten to permeative pattern and a wide zone of transition (poor margination). It is also common cortical destruction with a large associated soft-tissue mass, usually homogeneous in attenuation. Aggressive periosteal reaction is frequent, either lamellated (onion skin) or spiculated (sunburst or hair-on-end).^5^ MRI is the best modality in evaluation local staging of the mass, particularly for the assessment of focal extension and involvement of adjacent structures. In 96% of cases, ES is associated with a large soft-tissue mass that is often circumferential but asymmetric around the osseous involvement. The signal intensity is usually homogeneous and low to intermediate on T2-weighted images.^5,6^ On DWI, ES has marked low diffusion with ADC values of 0.6–0.7 × 10^−3^ mm^2^/s.^11,12^ Diffuse or peripheral nodular contrast enhancement is present in all cases of ES. Homogeneity and signal intensity are related to the high degree of cellularity in ES. Sparse heterogeneity is more common in larger lesions.^5^ ABC-like areas (previously called “secondary ABC-cyst”) can be seen in bone tumors that have undergone hemorrhagic cystic change. It is commonly associated with benign neoplasms such as giant cell tumor, osteoblastoma, chondroblastoma, fibrous dysplasia, and chondromyxoid fibroma, but it can also appear in malignant neoplasms, most commonly osteosarcoma (telangiectatic osteosarcoma).^2^

In our case, imaging features of ES had permeative bone destruction and aggressive periosteal reaction with spiculated pattern at CT. MRI showed a small homogeneous extracranial soft-tissue mass with signal intensity typical for tumors with high degree of cellularity, but the tumor had a large intracranial soft-tissue mass with mainly multi-cystic spaces with fluid-fluid levels that we have considered as ABC-like changes, extremely unusual in ES. To the best of our knowledge, no previous case of the radiological features of primary ES of the temporal bone with ABC-like areas has been published, except for one case in the sphenoid bone extending into the orbit and middle cranial fossa comprised of multi-cystic spaces with fluid–fluid levels.^13^

Definitive diagnosis of ES relies on biopsy, providing sufficient material for histology, immunohistochemistry, and molecular genetic testing. Histologically, ES is a small round cell sarcoma. Immunohistochemically, the tumors show a strong membranous positivity for CD99, but cytogenetic and molecular techniques are required to distinguish ES from other tumors. ES is associated with structural rearrangements that generate FET-ETS fusion genes, creating an aberrant transcription factor. The most common ES translocation is t (11;22) (q24;q12), which results in the EWSR1-FLI1 fusion transcript and protein.^2^

One of the first steps after a patient has been diagnosed ES is to determinate if there are metastasis as this impact both their future therapies as well as prognosis. The majority of ES initially have subclinical micrometastasis that will become apparent in the future if the patient does not receive systemic treatment, and approximately 25% of patients with ES have detectable metastatic disease at diagnosis. The metastatic workup includes ^18^F-FDG-PET/CT and/or whole-body MRI for detecting skeletal metastasis, and chest CT for staging lung metastasis.^14–17^

Several studies have proved the usefulness of DWI and dynamic contrast-enhanced MRI (DCE) to improve an accurate diagnosis, and for monitoring of response to treatment in bone and soft-tissue tumors, including ES. These functional sequences can been used to evaluate the decreased cellularity from reduced viable tumor and the degree of necrosis corresponding to a positive tumor response histologically.^18^ This is of relevance for early detection of treatment response and failure, as morphological parameters alone do not necessarily correlate with treatment response and prognosis.^18–23^ In our case, the follow-up MRI 2, and 4 months after the start of treatment showed full regression of extracranial soft-tissue component and partial regression of the intracranial soft-tissue component, indicating good treatment response. The remaining intracranial component had areas reminiscent of ABC-likes changes, no areas of low diffusion, and septal and rim contrast enhancement, indicating some viable residual tumor tissue.

Treatment of ES is usually a combination of neoadjuvant chemotherapy followed by surgical resection; in some cases, it can be supplemented with adjuvant chemotherapy or radiation therapy.^6–8^ The prognosis of ES has a 65–70% cure rate for localized disease, but metastatic and early-relapsing tumors have a poor prognosis, with a 5-year survival rate of <30%.^2^ Only 100% necrosis after neoadjuvant chemotherapy and surgery is a favorable prognostic factor.^24^ In this case, we used chemotherapy, radiotherapy, and radiology according to our institutional guidelines. This may not be suitable for all countries or for all patients where resources are limited. Nevertheless, the patient was treated according to well-known international guidelines.^25,26^

In summary, our case report presents an extremely rare radiological presentation of primary ES of temporal bone associated with large ABC-likes areas. Recognition of this atypical radiological feature might help radiologists to be aware of ES as a possible diagnosis. Tissue sampling is required for a definitive diagnosis. This paper also underlines the importance of a multidisciplinary management to achieve an accurate diagnosis, successful treatment, and good outcome of ES of cranial bones.
